# A Switch from Latent to Typical Infection during *Pectobacterium atrosepticum*—Tobacco Interactions: Predicted and True Molecular Players

**DOI:** 10.3390/ijms241713283

**Published:** 2023-08-27

**Authors:** Ivan Tsers, Olga Parfirova, Varvara Moruzhenkova, Olga Petrova, Natalia Gogoleva, Vladimir Vorob’ev, Yuri Gogolev, Vladimir Gorshkov

**Affiliations:** 1 Kazan Institute of Biochemistry and Biophysics, Federal Research Center “Kazan Scientific Center of the Russian Academy of Sciences”, 420111 Kazan, Russia; ivantsers@gmail.com (I.T.); parfirovaolga.i@gmail.com (O.P.); varvara.moruzhenkova@gmail.com (V.M.); poe60@mail.ru (O.P.); negogoleva@gmail.com (N.G.); vorobyev@kibb.knc.ru (V.V.); gogolev.yuri@gmail.com (Y.G.); 2 Institute of Fundamental Medicine and Biology, Kazan Federal University, 420008 Kazan, Russia

**Keywords:** ethylene, jasmonates, *Pectobacterium*, plant infectious diseases, plant susceptible responses, typical and latent infections

## Abstract

Phytopathogenic microorganisms, being able to cause plant diseases, usually interact with hosts asymptomatically, resulting in the development of latent infections. Knowledge of the mechanisms that trigger a switch from latent to typical, symptomatic infection is of great importance from the perspectives of both fundamental science and disease management. No studies to date have compared, at the systemic molecular level, the physiological portraits of plants when different infection types (typical and latent) are developed. The only phytopathogenic bacterium for which latent infections were not only widely described but also at least fluently characterized at the molecular level is *Pectobacterium atrosepticum* (*Pba*). The present study aimed at the comparison of plant transcriptome responses during typical and latent infections caused by *Pba* in order to identify and then experimentally verify the key molecular players that act as switchers, turning peaceful plant-*Pba* coexistence into a typical infection. Based on RNA-Seq, we predicted plant cell wall-, secondary metabolism-, and phytohormone-related genes whose products contributed to the development of the disease or provided asymptomatic plant—*Pba* interactions. By treatment tests, we confirmed that a switch from latent to typical *Pba*-caused infection is determined by the plant susceptible responses mediated by the joint action of ethylene and jasmonates.

## 1. Introduction

Most, if not all, phytopathogens do not obligatorily cause disease since they can interact with host plants asymptomatically for a long period and even throughout the whole life cycle of the host [1]. Knowledge of the mechanisms that trigger a switch from latent (asymptomatic) to a typical (symptomatic) infection and thus disturb the equilibrium between the host and pathogen is of great importance. The elucidation of these mechanisms will advance the fundamental understanding of pathosystem development and lay the groundwork for managing acute pathological processes in agricultural plants.

Unfortunately, latent, asymptomatic infections have received very little attention from researchers. Although a number of latent infections caused by various phytopathogens have been described [2,3,4], no studies have been performed to date to compare, at the systemic molecular level, the physiological portraits of plants when different infection types (typical and latent) caused by a particular pathogen are developed. The physiological bases for switching peaceful plant—pathogen coexistence to an acute pathological process are also unknown. Currently, due to a gap in understanding the switch from a latent to a typical infection, latent infections are objectively considered a big danger because we cannot control the transition from asymptomatic infection to symptomatic infection associated with yield losses or even forecast such a transition. However, if the markers of such a switch are unraveled and properly targeted using conventional breeding, genome editing techniques, or treatment approaches, latent infections will pose a much smaller threat to food security.

*Pectobacterium* species, due to their brute force related to the production of an arsenal of plant cell wall-degrading enzymes (PCWDEs), can cause extensive maceration of host plant tissues [5,6]. However, these bacteria have been widely shown to interact with both weed and crop plants asymptomatically for a prolonged period and even to be vertically transmitted to subsequent host generations without the manifestation of any disease symptoms [7,8,9,10,11,12]. Nevertheless, the plant—*Pectobacterium* equilibrium related to the latent infection can be disturbed, leading to the development of a typical infection associated with extensive rotting.

The *cfa6*-mutant of *Pectobacterium atrosepticum* (*Pba*), deficient in the production of coronafacic acid, a functional analog of the plant phytohormone jasmonic acid (JA), has been shown to systemically colonize tobacco plants without causing the disease in most cases [13]. Herewith, the mutant’s systemic, asymptomatic colonization of plants is limited to primary xylem vessels. In turn, the wild-type (WT) strain, causing mostly typical infections, in addition to primary xylem vessels, extensively colonizes the parenchyma, which is destroyed by the pathogen.

Within the primary vessels, both WT and *cfa6*-mutant form bacterial emboli—specific biofilm-like structures [14]. The major constituent of the primary extracellular matrix of bacterial emboli is the plant cell wall pectic polysaccharide rhamnogalacturonan I (RG-I). RG-I is released from plant cell walls into the primary vessel lumen where it is further used by bacteria as a structural component for assembling bacterial emboli [15,16]. Importantly, the release of RG-I starts at the very beginning stage of vessel colonization (or even prior to vessel colonization) when microbial PCWDEs are yet absent (at least at amounts sufficient to cause significant plant cell wall remodeling). Therefore, it is evident that the release of RG-I is provided by the host plant, but not bacterial gene products. These host gene products are recruited by *Pba* for its own needs. Such recruitments are referred to as susceptible responses—i.e., host reactions driven by pathogen manipulation in order to “improve” its ecological niche [17,18]. Particularly, pathogens can recruit the host’s plant cell wall enzymes and proteins (expansins) to facilitate plant cell wall loosening [19,20,21,22,23]. Various host plant susceptible responses can drive pathogen behavior and determine the outcome of the host—pathogen interaction, including whether a typical or latent infection develops [17,18].

Thus, typical and latent infections, caused by WT *Pba* and its *cfa6*-mutant, respectively, have common features (related to the colonization of the primary xylem vessels and plant responses leading to the release of RG-I) and distinctive features (related to the colonization of the parenchyma and the formation of rotting symptoms). We used these two infection types to compare pathosystems that developed according to different scenarios. Our study aimed to compare plant transcriptome responses during typical and latent infections in order to identify key molecular players that can act as switchers, turning peaceful plant—*Pba* coexistence into a typical symptomatic infection.

## 2. Results

### 2.1. Regulation of Gene Expression during Typical and Latent Infections

The results of RNA-Seq read processing, mapping, and quantification are presented in Table S1. In total, 17,898 and 2712 differentially expressed genes (DEGs) were revealed for typical infection (TI) and latent infection (LI), respectively, compared with control noninfected plants, whereas 13,926 genes were DEGs during LI compared with TI (Table 1). To interpret the obtained transcriptome profiles, the revealed DEGs were functionally split into hierarchical categories and subcategories based on the classification performed in our previous study [24] (Table 2 and Table S2). Below, we discuss the gene categories whose regulation seemed to have the greatest impact on the transition from LI to TI. These gene categories were enriched with DEGs (including those with the highest degree of regulation), and the physiological processes implemented by the gene products from these categories were previously considered from the viewpoint of their role in *Pectobacterium*-induced pathogenesis.

### 2.2. Plant Cell Wall Modification

**Figure 1:**
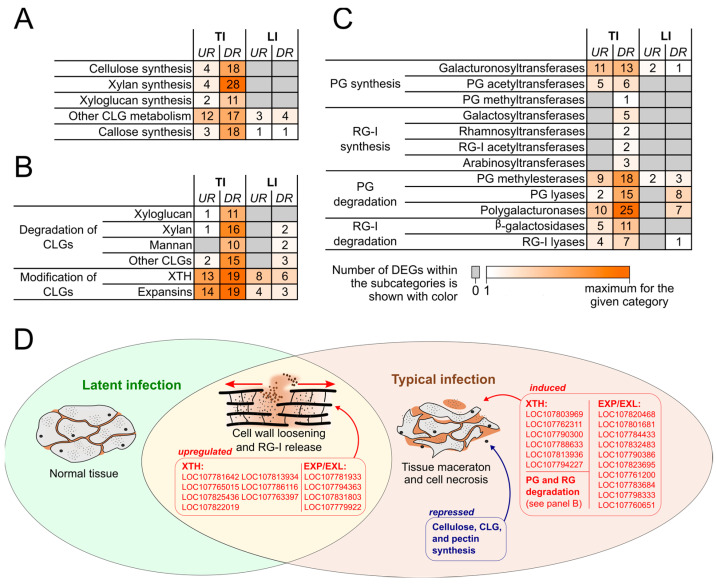
The regulation of expression of genes from the gene category “Plant cell wall modification” and the predicted consequence of their regulation in tobacco plants during the development of typical infection (TI) caused by the wild-type *Pectobacterium atrosepticum* SCRI1043 or during latent infection (LI) caused by the *cfa6*-mutant of *P. atrosepticum* compared with control noninfected plants. (**A**) Genes related to the syntheses of cellulose, callose, and cross-linking glycans (CLGs); (**B**) Genes related to the degradation of CLGs or modification of their bounds with cellulose; (**C**) Genes related to the synthesis and degradation of pectic compounds. Numbers in the panels show the quantities of upregulated “UR” and downregulated “DR” genes during TI or LI compared with noninfected plants. RG, rhamnogalacturonan; PG, polygalacturonan; XTH, xyloglucan endotransglucosylase/hydrolase; EXP/EXL, expansins and expansin-like proteins; (**D**) Schematic representation of the consequences of the regulation of the plant cell wall-related genes during TI and LI. The details of gene expression patterns are presented in Table S2.

Genes related to the synthesis of cellulose and callose as well as the synthesis and degradation of cross-linking glycans (CLGs) were mostly downregulated during TI, whereas most of these genes were non-DEGs during LI (Figure 1; Table S2). This means that the synthesis of cellulose, callose, and CLGs was repressed during TI but not during LI. A total of 19 encoding xyloglucan endotransglucosylase/hydrolases (XTH) and 19 genes encoding expansins were downregulated during TI (Figure 1; Table S2). However, 13 XTH-encoding genes and 14 expansin-encoding genes were upregulated during TI. Importantly, 7 TI-upregulated XTH-encoding genes (LOC107781642, LOC107765015, LOC107825436, LOC107822019, LOC107813934, LOC107786116, LOC107763397) and 4 TI-upregulated expansin-encoding genes (LOC107781933, LOC107794363, LOC107831803, LOC107779922, all encoding expansin-like proteins B) were also upregulated during LI (Figure 1; Table S2). Since XTH and expansins provide cell wall loosening and stretching, the products of those XTH- and expansin-encoding genes that were upregulated during both TI and LI seem to promote the release of RG-I into the vessels, occurring during both TI and LI. In turn, the products of 6 XTH-encoding genes and 10 expansin-encoding genes (6 expansins A, 3 expansin-like B proteins, and 1 expansin B) that were upregulated during TI but not during LI seem to facilitate maceration-associated cell wall loosening, contributing to the symptom development.

In our previous study, the upregulation of genes encoding pectin-degrading enzymes, including RG-I-lyases, was also presumed to contribute to the release of RG-I into vessels during TI [24]. However, in our present study, genes encoding enzymes that cleave glycosidic linkages in pectic compounds were upregulated only during TI, and none of them were upregulated during LI (Figure 1; Table S2). Given that RG-I release occurs during both TI and LI, the upregulation of genes encoding pectin-degrading enzymes only during TI seems to facilitate tissue maceration rather than the release of RG-I. Genes related to the synthesis of pectic compounds were mostly downregulated during TI and non-differentially expressed during LI.

### 2.3. Secondary Metabolism

**Figure 2:**
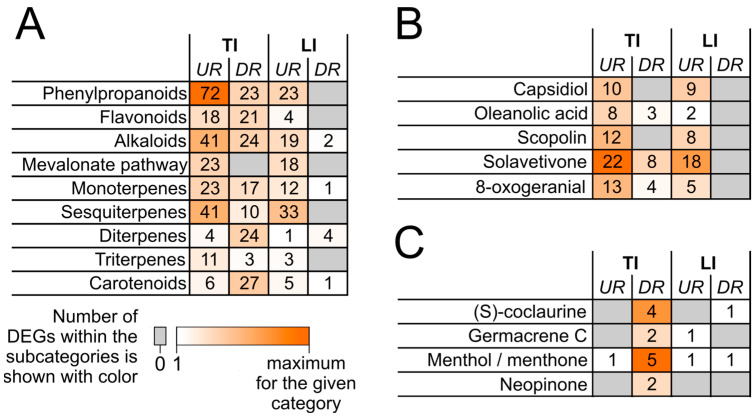
The number of genes from the subcategories of the category “Secondary metabolism” upregulated (UR) or downregulated (DR) during typical infection (TI) caused by the wild-type *Pectobacterium atrosepticum* SCRI1043 or during latent infection (LI) caused by the *cfa6*-mutant of *P. atrosepticum* compared with control noninfected plants. The details of the gene expression pattern are presented in Table S2. (**A**) A number of differentially expressed genes (DEGs) encoding proteins involved in the biosynthesis of phenylpropanoids, flavonoids, alkaloids, and terpenoids. (**B**) Subcategories of DEGs, for which the upregulation was more pronounced during TI than during LI. (**C**) Subcategories of DEGs, which were mostly downregulated during TI but not during LI.

During both TI and LI, genes related to the synthesis of phenylpropanoids, alkaloids, mono- and sesquiterpenes were mostly upregulated. Herewith, the number of such genes and the level of their upregulation were greater during TI than during LI (Figure 2; Table S2). This means that the corresponding secondary metabolites are unlikely to sustain LI and prevent it from progressing to TI. In turn, most genes related to the biosynthesis of diterpenoids and carotenoids were downregulated during TI but not during LI (Figure 2; Table S2). Thus, it may be presumed that the reduction of the levels of diterpenoids and carotenoids due to the downregulation of corresponding genes is a prerequisite for the transformation of LI into TI.

Considering the regulation of the expression of genes related to the synthesis of particular secondary metabolites, two major types of regulation were found. First, genes related to the synthesis of capsidiol, oleanolic acid, scopolin, solavetivon, and 8-oxogeranial were predominantly upregulated during both infection types, and during TI, these genes were upregulated more strongly than during LI (Figure 2; Table S2). This means that the resulting increase in the level of these metabolites hardly restricts the progression of disease symptoms. Second, genes related to the synthesis of (S)-coclaurine, germacrene C, menthol, and neopinine were downregulated during TI and non-differentially expressed during LI (Figure 2; Table S2). This means that the development of TI is likely to be coupled with a decrease in the levels of these secondary metabolites, whereas supporting their “initial” levels sustains LI. We found no secondary metabolites whose biosynthetic genes were upregulated (compared with control) more pronouncedly during LI than during TI.

### 2.4. Phytohormones

Our recent study showed that the lipoxygenase cascade, yielding the phytohormone jasmonic acid (JA) and many other oxylipins, is activated during TI compared with both control and LI [25]. The JA signal transduction pathway is also induced during TI compared with LI and control. This means that the progression of the *Pba*-caused disease is associated with the action of JA [25].

The opposite picture was observed in our present study for the JA antagonist, the phytohormone salicylic acid (SA). The SA-biosynthetic genes encoding isochorismate synthase were downregulated during TI but not during LI (Table S2). Genes related to the degradation of SA (DMR6-like oxygenase 2, [26]) were upregulated much more strongly during TI than during LI (Table S2). As a result, the SA-related marker genes PR-1 (LOC107763263, LOC107807833) [27] were upregulated during LI compared with TI (log_2_FC 6.7 and 3.0, respectively) (Table S2). This means that the relative JA-SA balance shifts toward JA during TI, while during LI, it shifts toward SA.

Genes related to the synthesis of the JA agonist, ethylene, were much more strongly upregulated during TI than during LI (Table S2). Ethylene signal transduction-related genes were upregulated only during TI. Genes of a large family of ethylene-responsive transcription factors (ERF) were much more strongly upregulated during TI (68 upregulated genes with log_2_FC > 5 for 27 of them) than during LI (13 upregulated genes with log_2_FC > 5 for 3 of them) (Table S2). Thus, ethylene, along with JA, is likely to play a role in the transformation of LI into TI.

Genes related to other phytohormones, such as auxin, gibberellins, cytokinins, and abscisic acid (ABA), were, in general, non-differentially expressed during LI. During TI, genes related to these phytohormones were found approximately equally among up- and downregulated genes, so it is difficult to interpret the alterations in the actions of these hormones during the infection. However, the majority of genes attributed to the subcategories “ABA synthesis”, “Auxin signaling”, and “Auxin transport” were downregulated during TI, indicating that the progression of the disease can be coupled with some repression of ABA- and auxin-mediated responses.

### 2.5. The Effect of JA and SA on Plant—Pba Interactions

**Figure 3:**
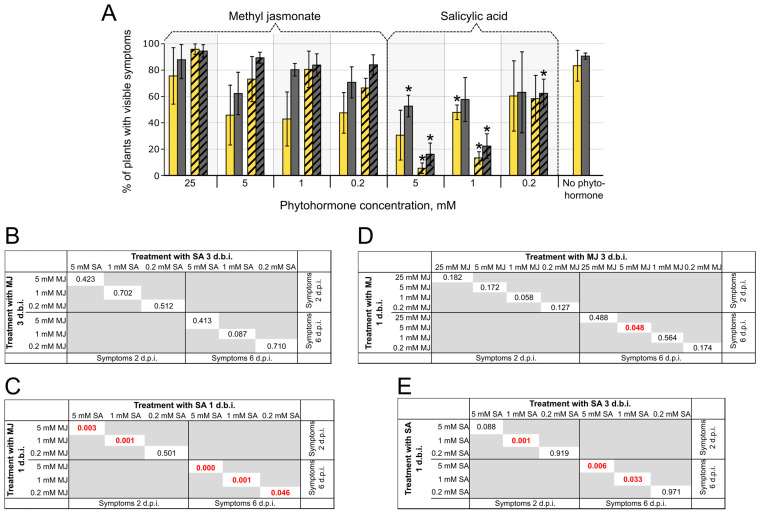
The effect of exogenous methyl jasmonate (MJ) and salicylic acid (SA) on the disease incidence rate in tobacco plants infected with *Pectobacterium atrosepticum* SCRI1043. (**A**) The percentage of plants with visible disease symptoms assessed on the 2nd (yellow) and 6th (gray) day postinoculation (d.p.i.) with *P. atrosepticum*. Plants were pretreated with MJ or SA for three days (solid columns) or one day (hatched columns) before inoculation (d.b.i.) with *P. atrosepticum*. Control—plants that were not treated with phytohormones before inoculation with *P. atrosepticum*. Asterisks (*) show the significance of the difference in the mean values between control (nontreated with phytohormones) plants and the indicated experimental group (two-sided *t*-test with Benjamini—Hochberg adjustment, FDR < 0.05). Other pairwise comparisons are presented in panels **B**–**E** as resulting *p*-values (two-sided *t*-test with Benjamini—Hochberg adjustment, FDR < 0.05): (**B**) plants treated with SA compared with plants treated with MJ three d.b.i.; (**C**) plants treated with SA compared with plants treated with MJ one d.b.i.; (**D**) plants treated with MJ three d.b.i. compared with one d.b.i.; (**E**) plants treated with SA three d.b.i. compared with one d.b.i. *p*-Values less than critical (*p* < 0.05) are marked in red. The presented results were obtained from three independent experiments; in each experiment, 20–25 plants were assessed for each experimental variant.

The above-described transcriptome analysis suggested that a shift in the JA-SA balance in favor of JA might promote the TI. To verify this, we analyzed the effect of methyl jasmonate (MJ) and SA on the disease caused by wild-type *Pba*. Since the action of phytohormones can be time-dependent [28,29,30], we differentially treated plants with different concentrations of MJ or SA three or one day before inoculation with *Pba*. The symptoms were assessed after two and six days postinfection. MJ at concentrations of 0.2–25 mM did not lead to statistically significant alterations in disease incidence after plant infection with *Pba* compared with MJ-nontreated plants (Figure 3). MJ concentrations greater than 25 mM resulted in yellowing and necrosis in noninfected plants. At 5 mM concentration, the MJ treatment three days before inoculation significantly reduced disease incidence compared with plants pretreated with 5 mM MJ one day before inoculation (Figure 3). This suggests that the increased level of MJ hardly restricts *Pba*-caused disease progression; however, it is possible that MJ can slightly reduce disease incidence when applied well in advance of infection.

In turn, the SA treatment at 1–5 mM concentrations significantly and strongly decreased disease incidence (Figure 3); higher SA concentrations led to yellowing and necrosis in noninfected plants. Interestingly, the treatment with SA one day before inoculation reduced disease incidence significantly more than the treatment with SA three days before inoculation (Figure 3). This shows that the protective effect of SA becomes less pronounced with time, while, in contrast, the protective effect of MJ slightly increases with time. Thus, SA inhibits the progression of *Pba*-caused disease, while the protective effect of MJ is only slightly visible (if any) when applied long before infection. This is in agreement with our transcriptome data showing that during TI and LI, the JA-SA balances are shifted in favor of JA and SA, respectively.

Since the *cfa6*-mutant strain, which causes LI that rarely (compared with wild-type-caused infections) transforms into TI, is deficient in coronafacic acid, a functional analog of JA, we hypothesized that MJ would restore the WT phenotype in the mutant strain, allowing it to cause TI at a high frequency. However, the treatment of plants with MJ did not increase the disease incidence caused by the mutant strain. This means that the artificial increase in the MJ level is not a sufficient condition to compensate for the inability of the mutant to synthesize coronafacic acid.

### 2.6. The Effect of Ethylene on Plant—Pba Interactions

Since MJ did not restore the *cfa6*-mutant’s ability to cause TI at a high incidence rate, we assumed that either another regulator(s) or MJ in conjunction with the other regulator(s) was involved in the transformation of LI into TI. Our transcriptomic data showed that, along with JA-related genes, genes related to ethylene were strongly upregulated during TI compared with both LI and noninfected plants (Table S2). Therefore, we assessed the effect of pretreatment with ethylene alone and ethylene together with MJ on the disease incidence rate caused by both WT *Pba* and its *cfa6*-deficient mutant.

**Figure 4:**
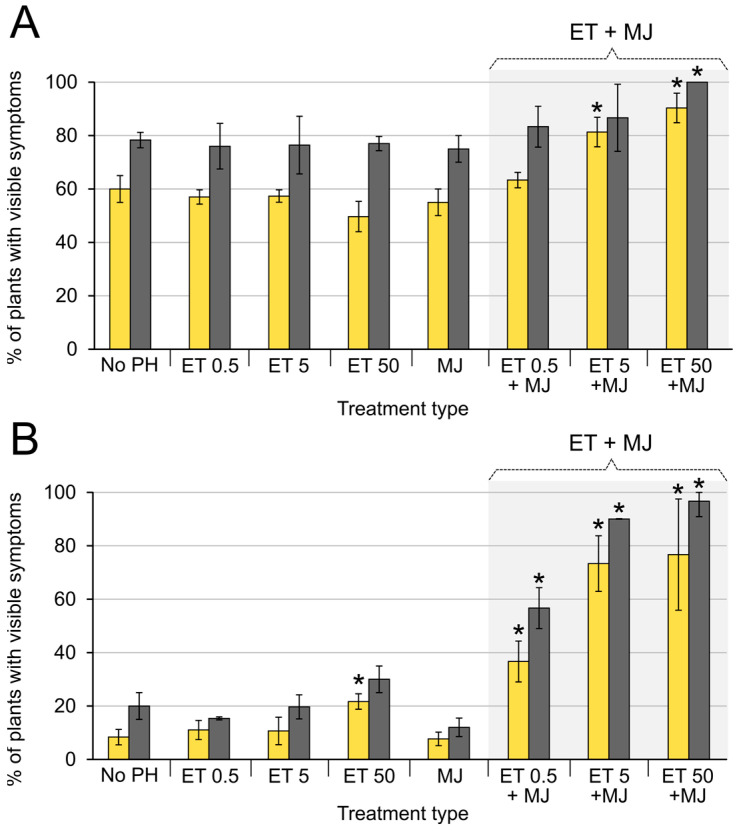
The effect of exogenous methyl jasmonate (MJ)and ethylene (ET), as well as ET and MJ together (ET + MJ) on the disease incidence rate in tobacco plants infected with *Pectobacterium atrosepticum* SCRI1043 (**A**) or its *cfa6*-mutant (**B**). The percentage of plants with visible disease symptoms was assessed on the 2nd (yellow) and 6th (gray) day postinoculation (d.p.i.) with bacteria. Plants were pretreated with phytohormones one day before inoculation (d.b.i.) with *P. atrosepticum*. MJ was applied at 1 mM concentration; ethylene was applied at concentrations of 0.5, 5, and 50 µL l^−1^. No PH (no phytohormones)—plants that were not treated with phytohormones before inoculation with bacteria. Asterisks (*) show the significance of the difference in the mean values between control (nontreated with phytohormones) plants and the indicated experimental group (two-sided *t*-test with Benjamini—Hochberg adjustment, FDR < 0.05). The presented results were obtained from three independent experiments; in each experiment, 20–25 plants were assessed for each experimental variant.

Exogenous MJ (1 mM) and ethylene (0.5, or 5, or 50 µL l^−1^) separately did not alter the disease incidence rate in both WT *Pba*- and *cfa6*-mutant-infected plants, except that ethylene at the highest concentration (50 µL l^−1^) slightly increased the percentage of symptomatic *cfa6*-mutant-infected plants at the first evaluation (two days postinfection) (Figure 4). The joint application of MJ and ethylene at higher ethylene concentrations (5 and 50 µL l^−1^) slightly increased disease incidence in plants infected with WT *Pba* (from 80% up to 100%). In turn, the application of MJ and ethylene together resulted in a dramatic increase in disease incidence in plants infected with the *cfa6*-mutant (from 20% up to 95%). Here, the disease incidence rate increased proportionally to the ethylene concentration (Figure 4).

**Figure 5:**
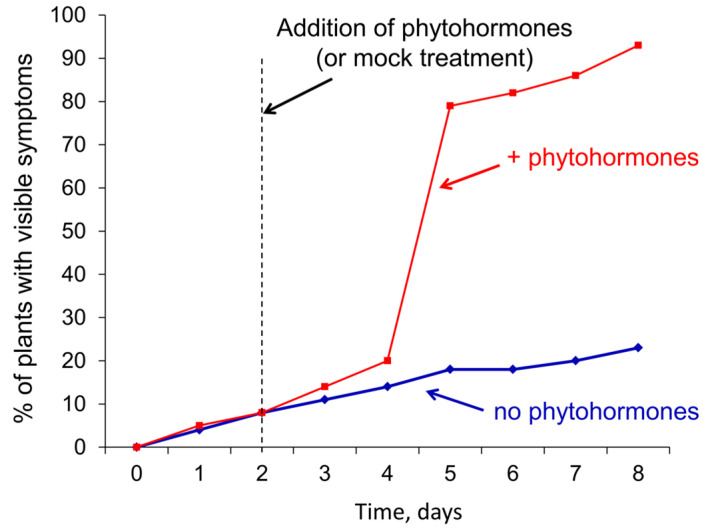
The effect of the postinfection treatment with methyl jasmonate (1 mM) and ethylene (5 µL l^−1^) on the dynamics of the disease incidence rate caused by the *cfa6*-mutant of *Pectobacterium atrosepticum* SCRI1043 in tobacco plants. Plants were nontreated with phytohormones (blue line) or treated with phytohormones (red line) two days after inoculation (vertical dotted line) with the *cfa6*-mutant. The presented values were obtained in one of two representative experiments; in each experiment, 100 plants were assayed for each experimental variant.

Then we checked whether postinfection treatment with MJ and ethylene also disturbed the equilibrium between plants and the *cfa6*-mutant and transformed LI into TI. Plants were infected with the mutant, and two days after inoculation, half of the plants were treated with phytohormones (1 mM MJ and 5 µL l^−1^ ethylene); another half of the plants remained untreated with phytohormones. Three days after the phytohormone application, a sharp transformation of LI to TI was observed (Figure 5). The disease incidence rate rose from 20% to 80% within a day (between the second and third day after the addition of phytohormones). In the phytohormone-nontreated variant, such a rise was not observed, and the level of disease incidence did not exceed 25% by the eighth day after infection (Figure 5). Thus, our results clearly demonstrate that the transformation of LI into TI is provided by the coordinated action of MJ and ethylene.

## 3. Discussion

Our study, for the first time, addresses the question of the mechanisms underlying the transformation of LI into TI during a plant—phytopathogen interaction. To answer this question, we compared two pathosystems: (1) tobacco plants infected with wild-type *Pba*, which in most cases causes TI related to extensive rotting of parenchymatous tissues, and (2) tobacco plants infected with the *cfa6*-mutant of *Pba*, which in most cases causes LI that is not associated with the degradation of the parenchyma and rarely transforms into TI [13].

Both studied infection types are associated with the colonization of primary xylem vessels where *Pba* cells form bacterial emboli. A prerequisite for the formation of bacterial emboli is a *Pba*-induced host plant reaction resulting in the release of the pectic polysaccharide RG-I from the plant cell wall into the vessel lumen where an extracellular matrix of RG-I consolidates individual bacterial cells into a holistic structure [15]. Such a host reaction can be regarded as one of the types of plant susceptible responses. In our recent review, we distinguished two major types of plant susceptible responses [17]. The susceptible responses of the first type improve pathogen fitness in planta without promoting symptom manifestation, whereas the susceptible responses of the second type facilitate symptom development. The release of RG-I evidently corresponds to the susceptible responses of the first type since it occurs during both LI and TI, whereas the susceptible responses of the second type remained unknown for plant—*Pectobacterium* interactions prior to our study.

By analyzing differentially expressed genes (DEGs), we highlighted three major functional categories (“Plant cell wall”, “Secondary metabolism”, and “Phytohormones”) of genes whose regulation seemed to contribute to the transformation of LI into TI. Within the “Plant cell wall” category, seven XTHs and four expansins B encoded by genes upregulated during both LI and TI (Figure 1) appeared as the most obvious candidates that provided the release of RG-I into the vessels. In turn, some other XTHs and expansins A and B, as well as pectin-degrading enzymes, all encoded by genes upregulated during TI but not during LI, seem to assist the pathogen’s enzymes in tissue maceration, leading to the transformation of LI into TI. Plant expansins and pectin-degrading enzymes have been previously shown to be recruited by pathogens to colonize hosts and cause diseases [19,20,21,22,23]. However, XTHs have not yet been described as susceptibility factors to pathogens, although the action of these enzymes may promote plant cell wall loosening, facilitating disease progression. Additionally, the repression of the biosynthesis of cellulose, callose, and CLGs due to the downregulation of corresponding genes during TI, but not during LI, may also facilitate symptom manifestation, contributing to the development of TI rather than LI (Figure 1). Taken together, the repression of the synthesis of plant cell wall polysaccharides, the activation of host-mediated degradation of polysaccharides by XTHs and pectin-degrading enzymes encoded by TI-upregulated genes, as well as the disruption of hydrogen bonds between CLGs and cellulose due to the action of TI-upregulated expansins, can induce the transformation of LI into TI during the plant—*Pba* interaction. In turn, the increase in the production of specific XTHs and expansins B during plant—*Pba* interactions is unlikely to be related to the development of disease symptoms but is likely to promote the release of RG-I into the vessel’s lumen, allowing for asymptomatic colonization of the primary xylem vessels.

No genes related to secondary metabolism were found to be more strongly upregulated during LI (compared with control) than during TI (compared with control). This means that the maintenance of LI cannot be explained by the de novo synthesis of some secondary metabolite(s) that suppress pathogen propagation or its aggressive behavior. In turn, many secondary metabolite-related genes were upregulated during TI more strongly than during LI, indicating that these metabolites may promote symptom development. Despite their widely discussed defense properties, plant secondary metabolites can be toxic to plant cells and may thus impede defense reactions, exacerbate plant cell death, and promote increased plant susceptibility to pathogens [31,32,33]. Moreover, plant secondary metabolites can induce virulence in several phytopathogens, including those closely related to *Pba Dickeya dadantii* [34]. Furthermore, *Pectobacterium* species have been shown to tolerate host plant secondary metabolites due to the presence of genes whose products can provide their detoxification [35,36,37]. Thus, the upregulation of secondary metabolite-related genes (at least many of them) seems to promote the transformation of LI into TI rather than represent an effective defense response against *Pba*.

Simultaneously, genes related to the synthesis of some secondary metabolites, such as ((S)-coclaurine, germacrene C, menthol, and neopinine), were downregulated during TI and non-differentially expressed during LI (Figure 2). This suggests that the transformation of LI into TI is coupled with the reduced synthesis of these metabolites, whereas their preinfection level can contribute to the maintenance of LI. This makes these secondary metabolites prime candidates for research into their potential protective properties against diseases caused by the *Pectobacterium* species.

Thus, based on RNA-Seq data, we predicted plant cell wall-related enzymes/proteins and secondary metabolites that are strong candidates to contribute to the determination of whether LI or TI would develop during the plant—*Pba* interaction. Importantly, the participation of these enzymes/proteins/metabolites in the target physiological process is consistent with what is known about *Pba*-induced plant cell wall modification and the effect of secondary metabolites on plant—*Pectobacterium* interactions. However, to definitely approve the role of these enzymes/proteins/metabolites in the switch from LI to TI and in *Pba*-induced pathogenesis in general, functional validation studies with specific model systems for each particular case should be performed.

Strong differences between LI and TI were observed regarding the expression of genes related to phytohormones that are typically considered from the perspective of their crucial regulatory role in mediating responses to pathogen invasion, namely SA, JA, and ethylene. In our previous study, we have shown that TI, but not LI, is coupled with strong activation of the lipoxygenase pathway, yielding the phytohormone JA and its derivatives as well as other physiologically active oxylipins [25]. In our present study, we showed that SA-related regulation was opposite to JA-related regulation during TI and LI. The SA-biosynthetic and SA-responsive genes were upregulated during LI compared with TI. This demonstrates that during LI and TI, the JA-SA balance shifts in favor of SA and JA, respectively. This is in accordance with the fact that the SA- and JA-regulated pathways are usually mutually antagonistic; thus, a plant prioritizes one of the two defense pathways (either SA- or JA-mediated) [38,39]. Several regulatory proteins that provide the antagonism of the SA- and JA-regulated pathways were identified (including SA-induced transcription factors such as WRKY70 and TGA, JA-regulated NAC transcription factors, MAP-kinase MPK4 [40,41,42,43,44,45,46]); however, the whole picture of the interaction of these two hormonal pathways is incomplete to date. In contrast to SA, ethylene is considered the phytohormone, which in most cases acts in agonism with JA during biotic stress [47]. The JA and ethylene pathways converge at the transcriptional activation of ERF1 transcription factors that, in turn, regulate the expression of a large number of genes responsive to both ethylene and JA [48]. The primary transcription factors of the ethylene-mediated signal transduction pathway (EIN3 and EIL1) are direct targets of JAZ proteins, crucial components of JA signaling [49], while EIL1 promotes JA synthesis by upregulating the expression of the lipoxygenase gene [50].

Pathogens typically exhibit differential susceptibility to SA- and JA/ethylene-mediated host responses, being highly susceptible to one and tolerant (or much less susceptible) to the other [51,52]. Due to this, many phytopathogens purposefully induce the plant defense system that is harmless to the pathogen, leading to repression of the system that is harmful to the pathogen [44,53,54]. The question of which phytohormonal pathway, either SA- or JA-mediated, is more effective against *Pectobacterium* species remains open. On the one hand, the treatment of calla lily plants with JA reduced *P. carotovorum*-caused symptoms more than treatment with the SA analog [55], and the JA-related *Arabidopsis thaliana* mutants were more susceptible to *P. carotovorum* than wild-type plants [56]. On the other hand, symptomatic *Pectobacterium* ssp.-caused infections are always associated with a strong upregulation of the JA pathway, which does not prevent disease development [13,24,25,55,57]. Additionally, JA serves as a chemoattractant for *P. brasiliense* [58]. Furthermore, the increase in plant resistance to *Pectobacterium* species due to various treatments is associated with a shift in the JA-SA balance in favor of SA [41,59,60,61]. Moreover, some *Pectobacterium* species synthesize a specific virulence factor, coronafacic acid, a functional analog of JA, indicating that these bacteria purposefully induce host JA responses [13,62]. Herewith, the repressors of the SA pathway were revealed among the extracellular metabolites of *P. carotovorum* [63]. These findings presumably suggest that *Pectobacterium*-induced activation of the JA pathways more often reflects the susceptible response than the resistant one and that this response is necessary to repress the SA-mediated defense reactions.

To check this hypothesis, we assessed the effect of exogenous MJ and SA on disease incidence caused by *Pba*. SA caused a strong reduction in the disease incidence rate, whereas MJ caused a slight (if any) effect (Figure 3). The observed effect of SA and JA on the development of *Pba*-caused disease explains the role of coronafacic acid in *Pba* virulence. Given that the *cfa6*-mutant is unable to produce coronafacic acid, we hypothesized that an exogenous MJ would restore the aggressive behavior typical of the WT in the mutant strain. However, the treatment of plants with MJ did not increase the disease incidence caused by the mutant, indicating that the artificial increase in MJ level is not a sufficient condition to compensate for the absence of coronafacic acid. Therefore, we presumed that JA may promote plant susceptibility to *Pba* only in conjunction with its agonist, ethylene, especially given that the ethylene-related genes were upregulated as strongly as the JA-related ones during TI compared with LI. Indeed, the combined treatment of plants with MJ and ethylene dramatically increased the disease incidence rate following infection with the *cfa6*-mutant, irrespective of whether phytohormones were applied before or after the infection (Figure 4 and Figure 5). Thus, our findings unequivocally demonstrate that the transition of latent *Pba*-caused infection into TI is provided by the joint action of jasmonates and ethylene. Therefore, the jasmonate- and ethylene-co-regulated reactions can be considered a susceptible response that facilitates *Pba*-caused symptom development. Interestingly, the joint action of these two phytohormones (jasmonates and ethylene) was also shown to increase plant susceptibility to the piercing-sucking insect pest (*Nilaparvata lugens*) [50].

Our study shows that plant tolerance to *Pba* (related to LI development) is determined by the relative “indifference” of a plant to the presence of the pathogen but not by the de novo synthesis of some defensive metabolites. In total, 6.6 times fewer genes were regulated during LI than during TI (Table 1), and the regulation of none of these genes (except for the SA-related genes) can be attributed to the increased level of defense metabolites during LI compared with TI. In turn, if a plant displays excessive sensitivity to the presence of a pathogen, as reflected in a global transcriptome rearrangement, the host-pathogen—equilibrium is disturbed, leading to disease progression. During plant—*Pba* interactions, coronafacic acid appears to promote such excessive sensitivity by activating JA- and ethylene-mediated responses. The mechanism by which coronafacic acid induces JA responses is evident since these two compounds are functional analogs. However, the mechanisms by which coronafacic acid triggers ethylene-mediated responses remain to be elucidated.

In fact, LI seem to be much more common during plant—pathogen interactions than TI, and thus, in most cases, the presence of a pathogen does not pose damage. However, LI can transform into TI at any time. Our study, in which the process of the transformation of LI into a TI was investigated for the first time, shows that this process is a consequence of the induction of specific host plant susceptible responses that lead to the disturbance of the host—pathogen equilibrium and the manifestation of disease symptoms. Deepening knowledge of the mechanisms of induction of such susceptible responses will form the basis for plant disease management. The external blockage of these responses can maintain plant fitness and productivity even if they are infected by pathogens.

In conclusion, the upregulation of reactions co-regulated by JA and ethylene disturbs the equilibrium between plants and *Pba*, and these two phytohormones are among those molecular players that switch LI into TI during plant—*Pba* interactions. Therefore, preventing the upregulation of these reactions following *Pba* invasion (using genome editing techniques or various treatments) seems to be a reasonable approach for *Pba*-caused disease management. An important question is whether JA and ethylene induce the transformation of LI into TI caused by other species of the genetically and phenotypically diverse *Pectobacterium* genus and, thus, whether such a disease management approach would be species-specific or relatively universal. In any case, the identification of reactions that transform LI into TI or restrict this transformation in different plant—pathogen interactions and finding approaches to control these reactions in the field is a promising way to prevent large yield losses due to plant infectious diseases.

Specific plant XTHs, expansins A and B, and pectin-degrading enzymes, as well as several plant secondary metabolites, also seem to participate in the development of *Pba*-caused disease; however, their role in this process needs further functional validation. In turn, (S)-coclaurine, germacrene C, menthol, and neopinine (or resembling compounds) are prime candidates to suppress the transformation of LI into TI during plant—*Pba* interactions, which deserve further investigation from the viewpoint of possible protective properties of these secondary metabolites against *Pba*.

The development of LI is associated with much less transcriptome rearrangement than TI; therefore, the maintenance of plant—pathogen equilibrium seems to be coupled with relative plant “insensitivity” to *Pba* colonization. In turn, excessive plant sensitivity to *Pba* colonization reflected in dramatic transcriptome rearrangements likely disturbs the host—pathogen equilibrium, resulting in extensive symptom development. Finally, we would like to mention that to get a better insight into the physiological reasons for plant infectious disease development, more attention should be paid to studying latent infections and comparing them with symptomatic ones.

## 4. Materials and Methods

### 4.1. Bacteria and Plant Growth Conditions, Plant Inoculation

*Pectobacterium atrosepticum* SCRI1043 (*Pba*) [64] and its coronafacic acid-deficient *cfa6*-mutant with a disrupted *cfa6* gene [13] were grown in lysogeny broth (LB) at 28 °C and 180 rpm. *Nicotiana tabacum* cv. Petit Havana SR1 plants were grown axenically in tubes in a growth chamber with a 16 h:8 h, light:dark cycle photoperiod. Seeds were surface-sterilized using bleach (0.8% active chlorine) and 1% sodium dodecyl sulfate for 30 min, washed seven times with sterile distilled water, then transferred to Murashige and Skoog medium (MS) in Petri dishes. Ten-day-old seedlings were transferred to individual sterile tubes containing MS. Four (for phytohormone treatment assays) or six (for transcriptome analysis) weeks after planting, plants were infected. Bacteria were grown until the early stationary phase, then washed with sterile 10 mM MgSO_4_, and resuspended in the same solution up to a density of ~2 × 10^7^ CFU mL^−1^. Sterile 10 mM MgSO_4_ (control) or bacterial suspensions containing ~2 × 10^5^ cells were placed as 10 μL drops into the bosoms of the leaves in the middle part of the stems (for transcriptome analysis) or on the leaf surface (for phytohormone treatment assays) using sterile pipette tips, and slight scratches were made simultaneously.

### 4.2. RNA Extraction and cDNA Library Preparation

Stem sections in the asymptomatic zone (0.5 cm below the symptomatic, macerated area formed at the point of inoculation) were taken from *Pba*-infected plants for RNA extraction two days after plant inoculation; the corresponding stem sections were taken from *cfa6*-mutant-infected and noninfected (control) plants. Plant material was ground in liquid nitrogen. The obtained powder was resuspended in 1 mL ExtractRNA Reagent (Evrogen, Moscow, Russia) and RNA was extracted according to the manufacturer’s instructions. RNA samples were treated with a DNA-free kit (Life Technologies, Carlsbad, CA, USA). RNA quantity and quality were analyzed using a Qubit fluorimeter (Life Technologies) and Qsep100 DNA Analyzer (Bioptic, New Taipei City, Taiwan), respectively. Total RNA (1 µg) was enriched by mRNA using the NEBNext Poly(A) mRNA Magnetic Isolation Module (NEB, Ipswich, MA, USA). cDNA libraries were prepared using the NEBNext Ultra II Directional RNA Library Prep Kit for Illumina (NEB) according to the manufacturer’s instructions. The quality and quantity of the cDNA libraries before sequencing were monitored using an Agilent 2100 Bioanalyzer (Agilent, Santa Clara, CA, USA) and a CFX96 Touch Real-Time PCR Detection System (Bio-Rad, Hercules, CA, USA). Libraries were sequenced in three biological replicates. Sequencing was conducted on an Illumina HiSeq 2500 (Illumina, San Diego, CA, USA) at the Joint KFU–Riken Laboratory, Kazan Federal University (Kazan, Russia).

### 4.3. Gene Expression Analysis

Raw reads generated in this study are available at the NCBI BioProject under the accession number PRJNA898126. The quality of the obtained RNA-Seq reads was assessed using the FastQC tool (http://www.bioinformatics.babraham.ac.uk/projects/fastqc/) (accessed on 7 March 2023). Reads with q-score < 30 and rRNA-corresponding reads were filtered out using Trimmomatic and SortMeRNA, respectively [65,66]. To create the optimal reference for read pseudo-alignment, the EvidentialGene package (https://sourceforge.net/projects/evidentialgene/) (accessed on 7 March 2023) was used with default parameters to reduce the redundancy of tobacco coding sequence (CDS) (NCBI Assembly GCF_000715135.1). Pseudo-alignment and quantification of filtered reads were carried out using kallisto [67] with default parameters. The edgeR package [68] was used to reveal differentially expressed genes (DEGs). Genes that had TMM-normalized read counts per million (CPM) values ≥ 1 in all replicates within at least one of the experimental conditions were considered to be expressed in our study. Genes with |log_2_ FC| > 1 and false discovery rate (FDR) < 0.05 were considered to be DEGs. The functional annotations were assigned to the DEGs as described earlier [24].

### 4.4. Phytohormone Treatment Assays

Plants were sprayed with 200 µL of salicylic acid (SA) (0.2, 1, or 5 mM), or methyl jasmonate (MJ) (0.2, 1, 5, or 25 mM), or treated with ethylene (0.5, 5, or 50 µL l^−1^). The SA and MJ solutions were sterilized by filtration through 0.2 µm pore sterile filters (Corning, Berlin, Germany). Distilled water was used for the control treatment. Ethylene treatment was performed in airtight transparent chambers of 100 L volume. After each chamber opening (for plant inoculation or disease scoring), ethylene was again added into the chamber. When treated with both MJ and ethylene, plants were first sprayed with MJ and then transferred to airtight chambers and treated with ethylene. Phytohormone treatment was performed three or one day before infection or two days after infection. The results of the preinfection phytohormone treatment were obtained from three independent experiments; in each experiment, 20–25 plants were assessed for each experimental variant. Two independent experiments with 100 assessed plants in each experimental variant were performed for postinfection treatment.

### 4.5. Statistical Analysis

The statistical analysis was performed using a two-sided *t*-test with Benjamini—Hochberg adjustment. The results with a false discovery rate less than the critical value of 0.05 (FDR < 0.05) were considered statistically significant.

## Figures and Tables

**Table 1:** Differentially expressed genes (DEGs) revealed for tobacco plants, noninfected (control) or infected with *Pectobacterium atrosepticum* (causing typical infection (TI)) or its *cfa6*-mutant (causing latent infection (LI)).

Comparison	Expressed	DEGs	Upregulated DEGs	Downregulated DEGs
TI vs. Control	32,404	17,898	7681	10,217
LI vs. Control	31,964	2712	1744	968
LI vs. TI	31,233	13,926	8242	5684

**Table 2:** Functional classification of differentially expressed genes (DEGs) revealed for tobacco plants, noninfected (control) or infected with *Pectobacterium atrosepticum* (causing typical infection (TI)) or its *cfa6*-mutant (causing latent infection (LI)). For details see Table S2. Up- and downregulated genes are marked by red and blue, respectively.

Functional Supercategory	Number of Categories (Subcategories If Applicable)	Number of DEGs in TI vs. Control (Up- and Downregulated)	Number of DEGs in LI vs. Control (Up- and Downregulated)	Number of DEGs in LI vs. TI (Up- and Downregulated)
Cell cycle	6	220 (29 and 191)	17 (2 and 15)	179 (156 and 23)
Plant cell wall modification	28 (60)	764 (232 and 532)	157 (75 and 82)	630 (451 and 179)
Cytoskeleton	16 (19)	371 (141 and 230)	45 (27 and 18)	304 (191 and 113)
Other	16 (19)	2726 (1139 and 1587)	326 (199 and 127)	2118 (1334 and 784)
Phytohormones	19 (60)	771 (371 and 400)	178 (96 and 82)	563 (298 and 265)
Primary metabolism	21 (92)	3450 (1462 and 1988)	395 (283 and 112)	2849 (1750 and 1099)
Secondary metabolism	6 (71)	452 (276 and 176)	151 (141 and 10)	363 (152 and 211)
Signaling	10 (69)	2471 (1289 and 1182)	418 (308 and 110)	1873 (930 and 943)
Stress	10 (44)	802 (352 and 450)	198 (141 and 57)	642 (368 and 274)
Transcription factors	52 (124)	1013 (433 and 580)	175 (99 and 76)	773 (451 and 322)
Transport	27	1038 (574 and 464)	209 (156 and 53)	787 (382 and 405)

## Data Availability

Raw reads generated in this study are available at the NCBI BioProject under the accession number PRJNA898126.

## Supplementary Materials

The following supporting information can be downloaded at: https://www.mdpi.com/article/10.3390/ijms241713283/s1.

## References

[1] Kado C.I. (2010). Asymptomatic and Latent Infections. Plant Bacteriology.

[2] Jarvis W.R. (1994). Latent Infections in the Pre- and Postharvest Environment. HortScience.

[3] Stergiopoulos I., Gordon T.R. (2014). Cryptic fungal infections: The hidden agenda of plant pathogens. Front. Plant Sci..

[4] Takahashi H., Fukuhara T., Kitazawa H., Kormelink R. (2019). Virus Latency and the Impact on Plants. Front. Microbiol..

[5] Charkowski A., Blanco C., Condemine G., Expert D., Franza T., Hayes C., Hugouvieux-Cotte-Pattat N., López Solanilla E., Low D., Moleleki L. (2012). The role of secretion systems and small molecules in soft-rot *Enterobacteriaceae* pathogenicity. Annu. Rev. Phytopathol..

[6] Van Gijsegem F., van der Wolf J.M., Toth I.K. (2021). Plant Diseases Caused by Dickeya and Pectobacterium Species.

[7] Lapwood D.H., Harris R.I. (1982). The spread of *Erwinia carotovora* subsp. *atroseptica* and subsp. *carotovora* from stem lesions and degenerating seed tubers to progeny tubers in soil. Potato Res..

[8] Hélias V., Andrivon D., Jouan B. (2000). Development of symptoms caused by *Erwinia carotovora* ssp. *atroseptica* under field conditions and their effects on the yield of individual potato plants. Plant Pathol..

[9] Pérombelon M.C.M. (2002). Potato diseases caused by soft rot erwinias: An overview of pathogenesis. Plant Pathol..

[10] Tsror L., Lebiush S., Erlich O., Ben-Daniel B., Van Der Wolf J. (2010). First report of latent infection of *Cyperus rotundus* caused by a biovar 3 *Dickeya* sp.(Syn. *Erwinia chrysanthemi*) in Israel. New Dis. Rep..

[11] Tsror L., Lebiush S., Erlich O., Galilov I., Chalupowicz L., Reuven M., Dror O., Manulis-Sasson S. (2019). First report of latent infection of *Malva nicaeensis* caused by *Pectobacterium carotovorum* subsp. *brasiliense* in Israel. New Dis. Rep..

[12] Fikowicz-Krosko J., Czajkowski R. (2018). Systemic colonization and expression of disease symptoms on bittersweet nightshade (*Solanum dulcamara*) infected with a GFP-tagged *Dickeya solani* IPO2222 (IPO2254). Plant Dis..

[13] Gorshkov V., Gubaev R., Petrova O., Daminova A., Gogoleva N., Ageeva M., Parfirova O., Prokchorchik M., Nikolaichik Y., Gogolev Y. (2018). Transcriptome profiling helps to identify potential and true molecular switches of stealth to brute force behavior in *Pectobacterium atrosepticum* during systemic colonization of tobacco plants. Eur. J. Plant Pathol..

[14] Gorshkov V., Daminova A., Ageeva M., Petrova O., Gogoleva N., Tarasova N., Gogolev Y. (2014). Dissociation of a population of *Pectobacterium atrosepticum* SCRI1043 in tobacco plants: Formation of bacterial emboli and dormant cells. Protoplasma.

[15] Gorshkov V.Y., Daminova A.G., Mikshina P.V., Petrova O., Ageeva M., Salnikov V., Gorshkova T., Gogolev Y. (2016). Pathogen-induced conditioning of the primary xylem vessels–a prerequisite for the formation of bacterial emboli by *Pectobacterium atrosepticum*. Plant Biol..

[16] Gorshkov V., Tsers I., Islamov B., Ageeva M., Gogoleva N., Mikshina P., Parfirova O., Gogoleva O., Petrova O., Gorshkova T. (2021). The Modification of plant cell wall polysaccharides in potato plants during *Pectobacterium atrosepticum*-caused infection. Plants.

[17] Gorshkov V., Tsers I. (2022). Plant susceptible responses: The underestimated side of plant–pathogen interactions. Biol. Rev..

[18] Gorshkov V., Parfirova O. (2023). Host plant physiological transformation and microbial population heterogeneity as important determinants of the Soft Rot *Pectobacteriaceae*–plant interactions. Semin. Cell Dev. Biol..

[19] Vogel J.P., Raab T.K., Schiff C., Somerville S.C. (2002). PMR6, a pectate lyase-like gene required for powdery mildew susceptibility in *Arabidopsis*. Plant Cell.

[20] Flors V., de la O Leyva M., Vicedo B., Finiti I., Real M.D., García-Agustín P., Bennett A.B., González-Bosch C. (2007). Absence of the endo-beta-1,4-glucanases Cel1 and Cel2 reduces susceptibility to *Botrytis cinerea* in tomato. Plant J..

[21] Kay S., Hahn S., Marois E., Hause G., Bonas U. (2007). A bacterial effector acts as a plant transcription factor and induces a cell size regulator. Science.

[22] Cantu D., Vicente A.R., Greve L.C., Dewey F.M., Bennett A.B., Labavitch J.M., Powell A.L.T. (2008). The intersection between cell wall disassembly, ripening, and fruit susceptibility to *Botrytis cinerea*. Proc. Natl. Acad. Sci. USA.

[23] Abuqamar S., Ajeb S., Sham A., Enan M.R., Iratni R. (2013). A mutation in the expansin-like A2 gene enhances resistance to necrotrophic fungi and hypersensitivity to abiotic stress in *Arabidopsis thaliana*. Mol. Plant Pathol..

[24] Tsers I., Gorshkov V., Gogoleva N., Parfirova O., Petrova O., Gogolev Y. (2020). Plant soft rot development and regulation from the viewpoint of transcriptomic profiling. Plants.

[25] Gorshkov V.Y., Toporkova Y.Y., Tsers I.D., Smirnova E.O., Ogorodnikova A.V., Gogoleva N.E., Parfirova O.I., Petrova O.E., Gogolev Y.V. (2022). Differential modulation of the lipoxygenase cascade during typical and latent *Pectobacterium atrosepticum* infections. Ann. Bot..

[26] Zeilmaker T., Ludwig N.R., Elberse J., Seidl M.F., Berke L., Van Doorn A., Schuurink R.C., Snel B., Van den Ackerveken G. (2015). DOWNY MILDEW RESISTANT 6 and DMR 6-LIKE OXYGENASE 1 are partially redundant but distinct suppressors of immunity in *Arabidopsis*. Plant J..

[27] Grüner R., Strompen G., Pfitzner A.J., Pfitzner U.M. (2003). Salicylic acid and the hypersensitive response initiate distinct signal transduction pathways in tobacco that converge on the as-1-like element of the PR-1a promoter. Eur. J. Biochem..

[28] Slade W.O., Ray W.K., Hildreth S.B., Winkel B.S.J., Helm R.F. (2017). Exogenous Auxin Elicits Changes in the *Arabidopsis thaliana* Root Proteome in a Time-Dependent Manner. Proteomes.

[29] Lee J., Choi B., Yun A., Son N., Ahn G., Cha J.Y., Kim W.Y., Hwan I. (2021). Long-term abscisic acid promotes golden2-like1 degradation through constitutive photomorphogenic 1 in a light intensity-dependent manner to suppress chloroplast development. Plant Cell Environ..

[30] Feng Y., Wang X., Du T., Shu Y., Tan F., Wang J. (2022). Effects of Salicylic Acid Concentration and Post-Treatment Time on the Direct and Systemic Chemical Defense Responses in Maize (*Zea mays* L.) Following Exogenous Foliar Application. Molecules.

[31] Glazener J.A., Van Etten H.D. (1978). Phytotoxicity of phaseollin to, and alteration of phaseollin by, cell suspension cultures of *Phaseolus vulgaris*. Phytopathology.

[32] Dixon R.A., Maxwell C.A., Ni W., Oommen A., Paiva N.L. (1994). Genetic manipulation of lignin and phenylpropanoid compounds involved in interactions with microorganisms. Genet. Eng. Plant Second. Metabol..

[33] Rogers E.E., Glazebrook J., Ausubel F.M. (1996). Mode of action of the *Arabidopsis thaliana* phytoalexin camalexin and its role in Arabidopsis-pathogen interactions. Mol. Plant Microbe Interact..

[34] Yang S., Peng Q., San Francisco M., Wang Y., Zeng Q., Yang C.H. (2008). Type III secretion system genes of *Dickeya dadantii* 3937 are induced by plant phenolic acids. PLoS ONE.

[35] Lee D.H., Lim J.A., Lee J., Roh E., Jung K., Choi M., Oh C., Ryu S., Yun J., Heu S. (2013). Characterization of genes required for the pathogenicity of *Pectobacterium carotovorum* subsp. *carotovorum* Pcc21 in Chinese cabbage. Microbiology.

[36] van den Bosch T.J., Tan K., Joachimiak A., Welte C.U. (2018). Functional profiling and crystal structures of isothiocyanate hydrolases found in gut-associated and plant-pathogenic bacteria. Appl. Environ. Microbiol..

[37] van den Bosch T.J., Niemi O., Welte C.U. (2020). Single gene enables plant pathogenic *Pectobacterium* to overcome host-specific chemical defence. Mol. Plant Pathol..

[38] Beckers G.J.M., Spoel S.H. (2006). Fine-tuning plant defence signalling: Salicylate versus jasmonate. Plant Biol..

[39] Halim V.A., Vess A., Scheel D., Rosahl S. (2006). The role of salicylic acid and jasmonic acid in pathogen defence. Plant Biol..

[40] Van Verk M.C., Bol J.F., Linthorst H.J.M. (2011). WRKY transcription factors involved in activation of SA biosynthesis genes. BMC Plant Biol..

[41] Li J., Brader G., Palva E.T. (2004). The WRKY70 Transcription Factor: A Node of Convergence for Jasmonate-Mediated and Salicylate-Mediated Signals in Plant Defense. Plant Cell.

[42] Li J., Brader G., Kariola T., Palva E.T. (2006). WRKY70 modulates the selection of signaling pathways in plant defense. Plant J..

[43] Li N., Han X., Feng D., Yuan D., Huang L.J. (2019). Signaling crosstalk between salicylic acid and ethylene/jasmonate in plant defense: Do we understand what they are whispering?. Int. J. Mol. Sci..

[44] Zheng X.Y., Spivey N.W., Zeng W., Liu P.P., Fu Z.Q., Klessig D.F., He S.Y., Dong X. (2012). Coronatine promotes Pseudomonas syringae virulence in plants by activating a signaling cascade that inhibits salicylic acid accumulation. Cell Host Microbe.

[45] Petersen M., Brodersen P., Naested H., Andreasson E., Lindhart U., Johansen B., Nielsen H.B., Lacy M., Austin M.J., Parker J.E. (2000). Arabidopsis MAP kinase 4 negatively regulates systemic acquired resistance. Cell.

[46] Brodersen P., Petersen M., Nielsen B.H., Zhu S., Newman M.A., Shokat K.M., Rietz S., Parker J., Mundy J. (2006). Arabidopsis MAP kinase 4 regulates salicylic acid-and jasmonic acid/ethylene-dependent responses via EDS1 and PAD4. Plant J..

[47] Shinshi H. (2008). Ethylene-regulated transcription and crosstalk with jasmonic acid. Plant Sci..

[48] Lorenzo O., Piqueras R., Sánchez-Serrano J.J., Solano R. (2003). ETHYLENE RESPONSE FACTOR1 integrates signals from ethylene and jasmonate pathways in plant defense. Plant Cell.

[49] Zhu Z., An F., Feng Y., Li P., Xue L., Jiang Z., Kim J.M., To T.K., Li W., Zhang X. (2011). Derepression of ethylene-stabilized transcription factors (EIN3/EIL1) mediates jasmonate and ethylene signaling synergy in *Arabidopsis*. Proc. Natl. Acad. Sci. USA.

[50] Ma F., Yang X., Shi Z., Miao X. (2020). Novel crosstalk between ethylene-and jasmonic acid-pathway responses to a piercing–sucking insect in rice. New Phytol..

[51] Zhao Y., Thilmony R., Bender C.L., Schaller A., He S.Y., Howe G.A. (2003). Virulence systems of *Pseudomonas syringae* pv. *Tomato* promote bacterial speck disease in tomato by targeting the jasmonate signaling pathway. Plant J..

[52] Rahman T.A.E., Oirdi M.E., Gonzalez-Lamothe R., Bouarab K. (2012). Necrotrophic pathogens use the salicylic acid signaling pathway to promote disease development in tomato. Mol. Plant Microbe Interact..

[53] Robert-Seilaniantz A., Grant M., Jones J.D.G. (2011). Hormone crosstalk in plant disease and defense: More than just jasmonate-salicylate antagonism. Annu. Rev. Phytopathol..

[54] Pieterse C.M.J., Van der Does D., Zamioudis C., Leon-Reyes A., Van Wees S.C.M. (2012). Hormonal modulation of plant immunity. Annu. Rev. Cell Dev. Biol..

[55] Luzzatto-Knaan T., Kerem Z., Doron-Faigenboim A., Yedidia I. (2014). Priming of protein expression in the defence response of *Zantedeschia aethiopica* to *Pectobacterium carotovorum*. Mol. Plant Pathol..

[56] Norman-Setterblad C., Vidal S., Palva E.T. (2000). Interacting signal pathways control defense gene expression in Arabidopsis in response to cell wall-degrading enzymes from *Erwinia carotovora*. Mol. Plant Microbe Interact..

[57] Montesano M., Brader G., Ponce D.E., León I., Palva E.T. (2005). Multiple defence signals induced by *Erwinia carotovora* ssp. *carotovora* elicitors in potato. Mol. Plant Pathol..

[58] Tanui C.K., Shyntum D.Y., Sedibane P.K., Bellieny-Rabelo D., Moleleki L.N. (2021). *Pectobacterium brasiliense* 1692 chemotactic responses and the role of methyl-accepting chemotactic proteins in ecological fitness. Front. Plant Sci..

[59] Kariola T., Brader G., Li J., Palva E.T. (2005). Chlorophyllase 1, a damage control enzyme, affects the balance between defense pathways in plants. Plant Cell.

[60] Kariola T., Brader G., Helenius E., Li J., Heino P., Palva E.T. (2006). EARLY RESPONSIVE TO DEHYDRATION 15, a negative regulator of abscisic acid responses in Arabidopsis. Plant Physiol..

[61] Park H.B., Lee B., Kloepper J.W., Ryu C.M. (2013). One shot-two pathogens blocked: Exposure of *Arabidopsis* to hexadecane, a long chain volatile organic compound, confers induced resistance against both *Pectobacterium carotovorum* and *Pseudomonas syringae*. Plant Signal. Behav..

[62] Panda P., Vanga B.R., Lu A., Fiers M., Fineran P.C., Butler R., Armstrong K., Ronson C.W., Pitman A.R. (2016). *Pectobacterium atrosepticum* and *Pectobacterium carotovorum* harbor distinct, independently acquired integrative and conjugative elements encoding coronafacic acid that enhance virulence on potato stems. Front. Microbiol..

[63] Vidal S., de León I.P., Denecke J., Palva E.T. (1997). Salicylic acid and the plant pathogen *Erwinia carotovora* induce defense genes via antagonistic pathways. Plant J..

[64] Bell K.S., Sebaihia M., Pritchard L., Holden M.T.G., Hyman L.J., Holeva M.C., Thomson N.R., Bentley S.D., Churcher L.J.C., Mungall K. (2004). Genome sequence of the enterobacterial phytopathogen *Erwinia carotovora* subsp. *atroseptica* and characterization of virulence factors. Proc. Natl. Acad. Sci. USA.

[65] Kopylova E., Noé L., Touzet H. (2012). SortMeRNA: Fast and accurate filtering of ribosomal RNAs in metatranscriptomic data. Bioinformatics.

[66] Bolger A.M., Lohse M., Usadel B. (2014). Trimmomatic: A flexible trimmer for Illumina sequence data. Bioinformatics.

[67] Bray N.L., Pimentel H., Melsted P., Pachter L. (2016). Near-optimal probabilistic RNA-seq quantification. Nat. Biotechnol..

[68] Robinson M.D., Oshlack A. (2010). A scaling normalization method for differential expression analysis of RNA-seq data. Genome Biol..

